# Cyclic Stability of Locking Plate Augmented with Intramedullary Polymethyl Methacrylate (PMMA) Strut Fixation for Osteoporotic Humeral Fractures: A Biomechanical Study

**DOI:** 10.3390/life13112110

**Published:** 2023-10-24

**Authors:** Chih-Kun Hsiao, Yen-Wei Chiu, Hao-Yuan Hsiao, Yi-Jung Tsai, Cheng-Hung Lee, Cheng-Yo Yen, Yuan-Kun Tu

**Affiliations:** 1 Department of Medical Research, E-Da Hospital, I-Shou University, Kaohsiung 824005, Taiwan; shiaujk@gmail.com (C.-K.H.); ed113046@edah.org.tw (Y.-W.C.); ed108805@edah.org.tw (Y.-J.T.); 2 Department of Orthopaedics, E-Da Hospital, I-Shou University, Kaohsiung 824005, Taiwan; m8571409@yahoo.com.tw; 3 Department of Orthopedics, Taichung Veterans General Hospital, Taichung 407219, Taiwan; 298f@vghtc.gov.tw

**Keywords:** PMMA, intramedullary strut, stability, osteoporosis

## Abstract

The locking plate may provide improved fixation in osteoporotic bone; however, it has been reported to fail due to varus collapse or screw perforation of the articular surface, especially in osteoporotic bone with medial cortex comminution. Using bone graft as an intramedullary strut together with plate fixation may result in a stronger construct. However, the drawbacks of bone grafts include limited supply, high cost, and infection risk. PMMA (so-called bone cement) has been widely used for implant fixation due to its good mechanical properties, fabricability, and biocompatibility. The risk of donor-site infection and the drawbacks of allografting may be overcome by considering PMMA struts as alternatives to fibular grafts for humeral intramedullary grafting surgeries. However, the potential effects of intramedullary PMMA strut on the dynamic behaviour of osteoporotic humerus fractures remain unclear. This study aimed to investigate the influence of an intramedullary PMMA strut on the stability of unstable proximal humeral fractures in an osteoporotic synthetic model. Two fixation techniques, a locking plate alone (non-strut group) and the same fixation augmented with an intramedullary PMMA strut (with-strut group), were cyclically tested in 20 artificial humeral models. Axially cyclic testing was performed to 450 N for 10,000 cycles, intercyclic motion, cumulated fragment migration, and residual deformation of the constructs were determined at periodic cyclic intervals, and the groups were compared. Results showed that adding an intramedullary PMMA strut could decrease 1.6 times intercyclic motion, 2 times cumulated fracture gap migration, and 1.8 times residual deformation from non-strut fixation. During cycling, neither screw pull-out, cut-through, nor implant failure was observed in the strut-augmented group. We concluded that the plate-strut mechanism could enhance the cyclic stability of the fixation and minimize the residual displacement of the fragment in treating osteoporotic proximal humeral unstable fractures. The PMMA strut has the potential to substitute donor bone and serve as an intramedullary support when used in combination with locking plate fixation. The intramedullary support with bone cement can be considered a solution in the treatment of osteoporotic proximal humeral fractures, especially when there is medial comminution.

## 1. Introduction

Proximal humeral fractures are common in patients over 65 or those with osteoporosis. Most fractures (approximately 80–85%) are minimally displaced and stable and, therefore, can be effectively managed without surgery [1,2]. Several surgical approaches, such as open reduction, internal fixation, interlocking nails, or external fixation, are available for fracture management in patients with healthy bones. Previous biomechanical studies have demonstrated that the locking plates may provide significant advantages in treating comminuted humeral fractures with higher stability and strength than traditional compression fixation methods [3,4,5,6]. Thus, the locking plate system has become one of the most popular techniques to treat proximal humerus fractures. Few clinical reports exist on the results of locking plate fixation of proximal humeral fractures, and short-term functional outcomes and complications, such as humeral head varus collapse or intra-articular screw penetration have been variable in patients with osteoporosis [7,8,9]. In vitro studies indicated that these complications arise from locking plates without medial column support [10,11,12,13,14,15,16,17]. Biomechanical studies show that locking plates with intramedullary fibular allograft fixation can enhance the stability of the construct and reduce the humeral head migration compared to locking plate fixation alone [18,19,20,21,22,23]. Our previous study found that an intramedullary cortical strut significantly improved the overall stability and strength of locking plate fixation. In cases of poor bone quality, locked screw subsidence led to greater migration and lower stability of the fixations. The mechanism of the locking plate combined with inlay support may provide higher rigidity, improving fixation stability [24]. However, the stability of proximal humeral fractures remains a challenge if osteoporosis or severe loss of bone stock is presented.

To improve postoperative stability of unstable humeral fractures, autografts or allografts are commonly used to enhance fixation stability. Autografts use bone graft tissue typically harvested from the patient’s iliac crest or fibula. Autografts are considered the ‘gold standard’ for small bone defect reconstruction due to their remarkable osteogenic characteristics, which aid in bone healing, modelling, and remodelling. Using patient tissue increases the success rate of fusion. The benefit of using own tissue is that it increases the rates of successful fusion; however, the selection of an ideal bone graft relies on several factors, such as tissue viability, defect size and graft geometry, biomechanical properties, compatibility, graft handling cost, ethical issues, biological characteristics, pain, and donor site morbidity as well as other risks such as blood loss, visceral injuries during harvesting, wound infection, and associated complications are some of the risks. Allografts use cleaned and processed (sterilised) donor bone to ensure recipient safety. However, the risks include host immune response, disease transmission, local bone resorption, delayed incorporation and high cost have been presented [16,20,21]. Although in Europe, there are strict donor selection criteria that are effective in reducing the risk of infection reduce the risk of infection due to allograft [25,26,27]; however, bone grafting still suffers from high medical costs and unavailability of donor sources. Therefore, synthetic and biologically based tissue engineering biomaterials or their substitutes may be considered alternative options.

Polymethyl methacrylate (PMMA) has been widely used for implant fixation in orthopaedic and trauma surgeries due to its good mechanical properties, fabricability, and biocompatibility [28,29,30,31,32,33,34,35]. The risk of donor-site infection and the drawbacks of allografting may be overcome by considering PMMA struts as alternatives to fibular grafts for humeral intramedullary grafting surgeries. However, the impact of intramedullary PMMA struts on the biomechanical properties of osteoporotic humeral fractures remains uncertain. Therefore, we conducted the present study to investigate the effect of the intramedullary PMMA strut on the dynamic stability of osteoporotic proximal humeral fracture fixation.

## 2. Materials and Methods

### 2.1. PMMA Intramedullary Strut

**Figure 1:**
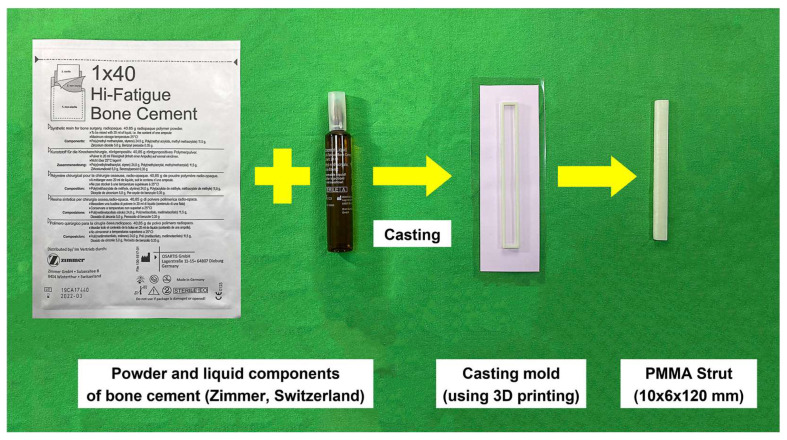
Powered MMA–styrene co-polymer (bone cement) and liquid MMA monomer are mixed to cast the prismatic-shaped PMMA strut (dimension: 10 × 6 × 120 mm).

PMMA (so-called bone cement) is an acrylic polymer material compounded by liquid MMA monomer and powdered MMA-styrene co-polymer [28,29]; the two components are mixed to form hardened PMMA. The liquid component includes methyl methacrylate 19.5 mL, N-Dimethyl-p-toluidine 0.5 mL (to prevent early polymerization), and hydroquinone 19.5 mg. The powder component includes methyl methacrylate styrene copolymer 30.0 g, polymethyl methacrylate 6.0 g, and barium sulfate 4.0 g. A three-dimensional printer (Sonic Mighty 8K, Phrozen, Hsinchu, Taiwan), using the stereolithography (SLA) technique, was applied to fabricate a groove mould (ABS-like cream) for casting the PMMA strut with the dimension of 10 × 6 × 120 mm. The bone cement was cast into the mould to form the PMMA strut. After polymerization, the bone cement strut was separated from the mould. The dimension of the intramedullary PMMA strut (10 × 6 × 120 mm) is based on the canal of the humeral shaft and our previous study [24]. Clinically, the dimension of the PMMA strut can be determined by the X-ray (C-arm) image and can be intraoperatively made in the operating room to fit the humeral canals of individual patients. Figure 1 depicts the powder and liquid components of bone cement, casting mould (3D printing), and the PMMA strut.

### 2.2. Artificial Bone Model, Implants and Fixations

Investigating the biomechanics of implants in osteoporotic bone poses several challenges because the human bone has interindividual variations, is not easily available in large quantities, and is expensive [30]. Therefore, twenty synthetic bones (Sawbones, Osteoporotic Humerus #1028-130, Vashon, Washington, DC, USA) were used in this study. These artificial bones were produced from two-component polyurethane foam (H75, VossChemie GmbH, Uetersen, Germany), comprising an osteoporotic-like trabecular network inside and a rigid layer replicating cortical bone.

**Figure 2:**
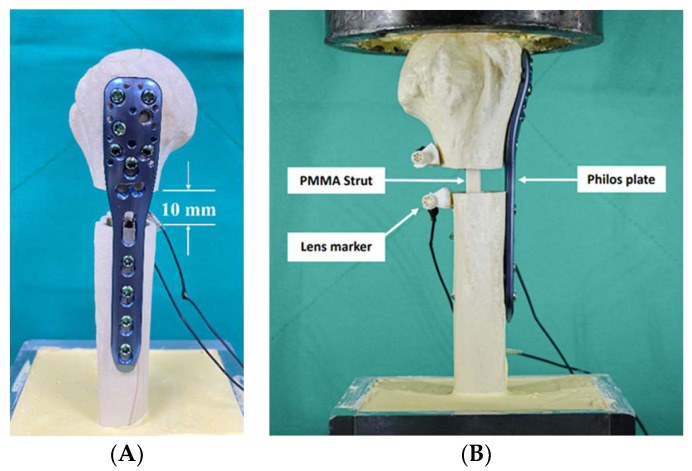
(**A**) Philos plate was fixed using locking screws and a 10 mm osteotomy gap was created to simulate the comminuted (unstable) fracture; (**B**) LED lens markers, PMMA strut, and experimental setup.

These synthetic bones were divided into two groups of ten specimens each. Two fixation techniques, non-augmented (or non-strut) and augmented (with strut), were tested. To simulate the proximal humeral comminuted (unstable) fracture, a 10 mm osteotomy gap was created at the level of the surgical neck. The fractures were stabilized using a locking plate (Philos, DePuy Synthes, Zuchwil, Switzerland) alone for non-strut construct. Pre-drilled screw holes using 2.8 mm drill bits were placed at the near cortex. Ten 3.5 mm locking screws (six for the proximal head and four for the distal shaft) were used for fixation. In the augmented group, the same plate (Philos) was used and combined with a 12 cm long PMMA intramedullary strut for fixation. The PMMA strut was placed into the humeral canal from the fracture site, and four locking screws were performed at the distal shaft and passed through the intramedullary strut. The strut was then retrograded up to the subchondral region of the humeral head, and then the head fragment was fixed with six screws. All constructs were resected at 20 cm from the mid-shaft of the synthetic specimens. The distal part of the construct was embedded and fixed in a custom-designed stainless-steel fixture using high-strength cement (Sakura New Hi-Stone, Yoshino Gypsum Co., Tokyo, Japan). At the proximal part of the construct, butter oil was smeared on the humeral head and partially immersed into the high-strength cement to create a cup-like cavity to simulate the glenoid fossa. The cement cup may contribute to an even distribution of the load transfer from the material testing machine to the humeral head. The cement cup was mounted on top of the jig and then the construct was vertically installed on the testing machine for testing. The PTI Visualeyez II VZ4000 motion tracking system (Phoenix Technologies, Incorporated, Vancouver, BC, Canada) was used to track the relative movements of the lens markers for determining the change of the osteotomy gap during cycling (accuracy = 0.015 mm). Two LED lens markers were set at each side of the far cortex of the osteotomy gap. The reference coordinate system was defined on the stainless-steel fixture (test platform). During testing, all kinematic data were recorded at a sampling rate of 100 Hz. The osteotomy gap, Philos plate and position of the screw, lens markers, PMMA strut and the experimental setup are presented in Figure 2.

### 2.3. Mechanical Testing

Cyclic testing was performed using an electrodynamic materials testing machine (Instron ElectroPuls E3000, INSTRON, Norwood, MA, USA). A sinusoidal waveform axial load with an amplitude of 450 N at a frequency of 1 Hz was applied on the top of the humeral head for 10,000 cycles. Load and kinematic data were continuously recorded during cycling. For more stable readings, the first 99 cycles were taken as preconditioning cycles, and the 100th cycle was defined as the initial cycle for comparison with the following cycles. The study evaluated the following loading protocol and parameters for comparison:

- Intercyclic motion: fragment gap amplitude (peak-to-peak displacement in 1 cycle) at 100, 1000, 2000, 4000, 6000, 8000 and 10,000 cycles;
- Cumulated fragment migration: the total change in gap distance at 450 N loading condition from the initial cycle to each 1000-cycle interval up to 10,000 cycles;
- Residual osteotomy gap deformation: cumulated plastic deformation at osteotomy gap from 100 to 10,000 cycles in the unloaded condition.

The loading protocol employed in this study was to simulate postoperative functional therapy for 6–8 weeks and replicate the daily activities. The rationale of this protocol was to create a more realistic testing environment and better understand the construct’s performance during the initial recovery period [31,32].

### 2.4. Statistical Analysis

Intercyclic motion (peak-to-peak displacement), fragment migration (displacement), and residual osteotomy gap deformation were presented as the mean and standard deviation. Significance analysis was performed using the Student’s *t*-test (normal distribution). Microsoft Excel (Microsoft Office Pro Excel 2021) was used for all statistical analyses. The statistical significance was set at *p* < 0.05.

## 3. Results

**Figure 3:**
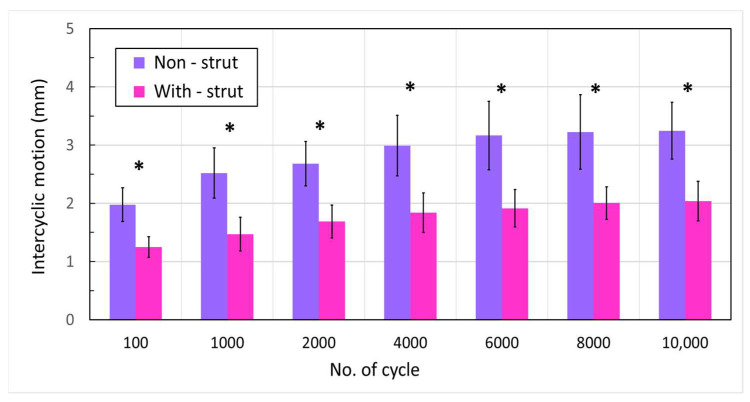
Intercyclic motion at 100, 1000, 2000, and each 2000-cycle interval up to 10,000 loading cycles (* *p* < 0.001).

The experimental results showed that the PMMA strut augmented group exhibited significantly less intercyclic motion than the non-augmented group (Figure 3). Specifically, mean intercyclic motion (peak-to-peak gap displacement) values ranged from 1.25 to 2.04 mm in the with-strut group, while they increased from 1.98 to 3.25 mm in the non-strut group. On average, the peak-to-peak gap displacement of the PMMA strut augmented group was 1.62 (1.62 ± 0.05) times less than that of the non-strut group (*p* < 0.001).

**Figure 4:**
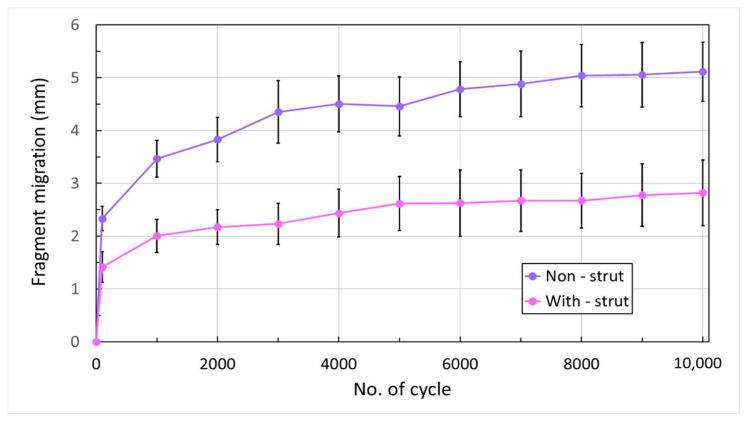
Maximum fragment migration (mean ± SD) at 100 and each 1000-cycle interval up to 10,000 loading cycles (*p* < 0.001 for all presented cycles).

The measured fragment migration between 100 to 10,000 cycles under 450 N loading increased from 2.3 to 5.1 mm for the non-strut group and 1.4 to 2.7 mm for the PMMA strut group (Figure 4). The fragment migration values of the with-strut (augmented with PMMA strut) group demonstrated approximately 2 times (1.99 ± 0.18) lower than that of the non-strut group. Moreover, the total change in gap distance under 450 N load was significantly less in the PMMA strut group than in the non-strut group at all time intervals up to 10,000 cycles (*p* < 0.05).

**Figure 5:**
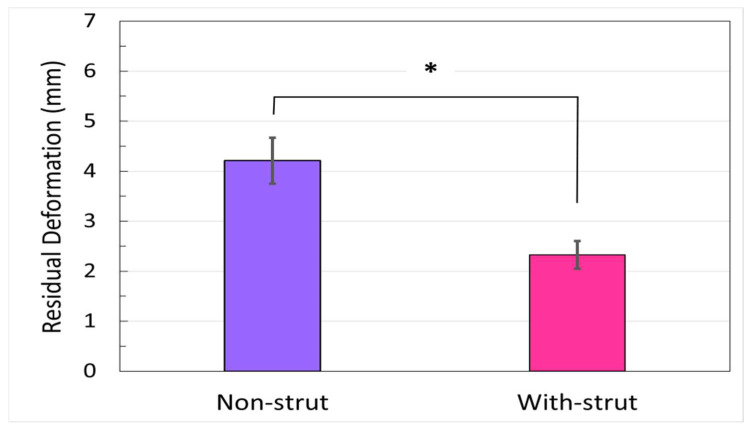
Residual deformation of the osteotomy gap (mean ± SD). The fragment gap distance from the initial (100th cycle) to the 10,000 loading cycle in the unloaded condition (* *p* < 0.001).

Figure 5 illustrates the residual deformation of the osteotomy gap after 10,000 loading cycles for two groups (mean ± SD). The cumulative plastic deformation (residual deformation) in the non-strut group was approximately 1.8 times (1.83 ± 0.26) higher than in the augmented group (4.21 ± 0.46 mm vs. 2.32 ± 0.28 mm; *p* < 0.001).

## 4. Discussion

Fibular grafts used in clinical practice can improve stability and reduce non-union rates in unstable proximal humerus fractures. However, the fibular is limited due to geometrical size-matching issues between the graft strut and medullary canals. Although we did not investigate the impact of strut length on interfragmentary motion in our study, a biomechanical study demonstrated no differences in interfragmentary movements when using struts with different lengths [36]. Thus, the length of the intramedullary strut used in our experiment was appropriate for augmenting the stability of the humeral head. Additionally, the position of the strut plays a crucial role in enhancing construct stability. Positioning the intramedullary strut closer to the medial side of the canal may increase the inlay support of the fracture, thus offering greater resistance to the varus moment.

Although locking plate fixation is a preferred treatment option for proximal humeral fractures, it has a high failure rate in elders or patients with osteoporosis. Osteoporotic bone has a weaker mechanical structure, creating challenges for achieving stable fixation of humeral fractures. Locking plates rely on fixed-angular screws to provide cantilever support for resisting varus collapse of the humeral head fragment. As a varus moment is applied, high stresses occur at the tips of the locked screws. The cyclic varus moment leads to repetitive axial loading and high compressive stress on the locking screws, causing an impact on cancellous bone and resulting in screw subsidence, which may lead to varus deformation, loss of reduction, and screw cut-out or perforation [7,8,9,12,15,37]. This phenomenon has been attributed to the stiffness of the construct which has been confirmed in a biomechanical study [37]. Anatomic reduction of the medial cortex is preferable and provides a stable intramedullary support column to create a load-sharing situation and minimize forces at the screw–bone interface. Previous studies also supported these findings, highlighting the need for improved fixation strategies in osteoporotic bone [12,37,38,39]. It seems from our results that the use of PMMA intramedullary strut may be one of the approaches to counteract these forces. Our study ensured that both groups were matched for osteoporosis and used the same locking plate (Philos) and screws. We hypothesised that adding a PMMA strut to the locking plate construct would enhance stability and reduce osteotomy gap migration compared to the locking plate alone. Our findings confirmed that the augmented groups exhibited significantly lower deformation and greater fixation stability than the non-strut groups. This finding highlights the ability of the intramedullary PMMA strut to provide additional support within the medullary canal, thus decreasing the load on the locked screw and minimising the risk of humeral head varus and screw cut-out. Our results are consistent with previous biomechanical studies and support the potential clinical benefits of using PMMA struts in this context [17,18,19,20,21,22].

To simulate the clinical scenario with no medial support in a proximal humerus comminuted fracture, a 10 mm osteotomy gap was created under the specimens’ greater tuberosity (surgical neck). Although the effect of the relative displacement of the fractural sides on the healing process remains unclear, Kralinger et al. [40] reported that the non-union rate was more frequent if the fracture was disturbed by motion during the healing period. In our study, we set a criterion of 3 mm loss of reduction for the relative motion of constructs between fragments. Our results showed that the non-strut group reached 3 mm of overall migration at 500 cycles; however, in the augmented group, the mean values of migration remained within 3 mm until 10,000 cycles (Figure 4). The PMMA strut-augmented group provides higher stability than the non-strut group. Zettl et al. [41] conducted a biomechanical study to compare the cyclic stability of monoaxial head locking screws and polyaxial locking screw systems under a 450 N load. Their results presented the benefit of the polyaxial locking screw systems. Praagman et al. suggested that the maximum compression across the glenohumeral joint at 90° of elevation was approximately 400 N [42], while Laursen et al. [43] reported a maximum force of less than 500 N across the same joint. Our study showed that the PMMA strut could provide sufficient short-term stability to withstand a 450 N axial load. However, we recommend not exceeding a 450 N load during the post-operative recovery period to prevent loss of reduction and implant failure.

It is unclear whether filling all screw holes in the plate would reduce the cut-out rate; however, it is reasonable to assume that adding more screws could increase the stability of the fixation, but the effect of the multiple screws on fracture-healing and the perfusion of the humeral head is not known. The current study used a fixation technique that involved six locked screws at the humeral head and three at the shaft. Despite the locked screw compressing the cancellous bone like a cantilever beam, microdamage still occurs in the cancellous bone, leading to humeral head migration. However, inserting the locked screw into the intramedullary strut allows the strut to provide inlay support at the humeral head and reduce subsidence. A finite element study has reported the biomechanical benefit of screw tip augmentation with bone cement [39]. In our augmented constructs, one locked screw was passed through the proximal portion and four were into the distal portion of the PMMA strut, providing additional screw purchase from the intramedullary strut and preventing implant loosening or screw cut-out at the humeral head fragment. Therefore, the plate–screw–strut mechanism can be seen as an effective stabilization. However, we recommend additional studies to investigate the optimal number and distribution of screws in the plate and further enhance the construct’s stability.

Comparing our results with previous studies is challenging due to the variations in loading protocols, fracture patterns, experimental setups and implant types used in each study. Our study used commercially available locking plates and screws. We emphasise the role of the PMMA intramedullary struts in treating osteoporotic proximal humeral fractures using locking plates. Our fixation technique is similar to the innovative fixation approach demonstrated by Brianza et al., wherein they used a specialised locking plate combined with a nail to enhance the interfragmentary stability [23]. Beirer et al. developed a blade device with a locking plate to reduce varus displacement of the humeral head, increase bone purchase, and improve the stability of the calcar region. Their technique could be further optimised by incorporating an intramedullary strut to provide additional support and decrease the risk of implant failure [44].

Clinically, antibiotics, such as gentamycin, tobramycin, erythromycin, cefuroxime, vancomycin and colistin are commonly used to treat local infections of bone and soft tissue and have improved the cure rate of infections. Bone cement has demonstrated its considerable utility due to its ability to incorporate specific active substances into the powder component. This transforms bone cement into a modern drug delivery system, enabling the targeted administration of necessary medications directly to the surgical site. Lautenschlager et al. [45] have established that the addition of various antibiotics to bone cement, in quantities below 2 g per standard packet (60 g) of bone cement, does not adversely affect certain mechanical properties of the cement, such as compressive or tensile strengths. However, quantities exceeding 2 g have been shown to weaken these properties. Studies also discovered that the inclusion of gentamicin at a concentration of 2/60 g per cement did not bring about any significant changes in compressive or tensile strength when compared to the control PMMA. Yang et al. reported that when bone cement and vancomycin were mixed manually in a low concentration (<5%) did not influence the strength of the bone cement [46,47,48]. In the presented study, no antibiotics were impregnated into the cemented strut. However, the effects of antibiotics on the biomechanical properties of bone grafts should be further investigated.

Proximal humeral fractures pose challenges in orthopaedic surgery due to their complex anatomy and variability in fracture patterns. Several fixation methods have been proposed, but there is no consensus on the optimal approach. Some titanium alloy commercial nailing systems have been used for treating humeral head fractures. Humeral nail designs have undergone significant innovation and can provide stable fixation in the humeral shaft distally as well as improved stability in the head and tuberosity fragments, which were the common site of fixation failure with earlier generation implants. Some studies have reported that the intramedullary nailing system is superior to the locking plate in reducing the total complication, intraoperative blood loss, operative time, postoperative fracture healing time and postoperative humeral head necrosis rate of proximal humerus fractures; however, the disadvantages of the humeral nailing systems include technical and intraoperative complications such as reaming complications and residual intraoperative malalignment [49,50]. Our study found that adding the PMMA strut provided medial column support and increased fixation stability, similar to the anteromedially placed helical blade reported by other researchers.

In some countries, bone cement may be off-label use; however, bone cement is still widely used in orthopaedic surgeries such as spine compression fracture surgeries (vertebroplasty, percutaneous balloon kyphoplasty), joint reconstruction, and trauma surgeries. In our study, we suggested that the PMMA strut may be used to substitute donor bone and serve as an intramedullary support when used in combination with locking plate fixation. This study provides important insights into the mechanical mechanism of the locking plate/intramedullary strut and its potential to improve the stability and clinical outcomes of humeral fracture fixation. Therefore, the intramedullary support with PMMA strut can be considered a solution in the treatment of osteoporotic proximal humeral unstable fractures. Although the combined locking plate–PMMA intramedullary strut implant may be an effective and simple treatment option for unstable proximal humerus fractures; however, further research is necessary to investigate the biomechanical properties of PMMA as an orthopaedic restorative material and its potential for use in other clinical scenarios.

This study has several limitations. First, we used an osteotomy gap to represent the fracture, which may not fully capture the complex fracture patterns commonly encountered in clinical settings. Second, while the bone density was consistent between specimens, the mechanical properties of the artificial bone used in the study may not fully represent those of human bone. Therefore, future research using human bone specimens is necessary for a better understanding of the mechanical behaviour of the fixation. Third, the dynamic testing protocols only examined the effects of axial loading transferable to the in vivo situation, the complete sequence of upper arm motion could not be simulated; thus, future investigations should also consider bending and torsional forces. Lastly, it is noteworthy that the study did not directly compare the PMMA augmentation technique with other surgical methods, such as intramedullary nailing or bone grafting. Further research is needed to compare the clinical outcomes of various surgical approaches for proximal humeral fractures and determine the most effective treatment strategy.

## 5. Conclusions

PMMA intramedullary strut in conjunction with a locking plate fixation significantly enhanced the construct’s stability, reducing intercyclic motion, osteotomy gap migration and residual gap deformation compared to the locking plate fixation alone. This study suggests that the combined locking plate–PMMA intramedullary strut implant may be an effective and simple treatment option for unstable proximal humerus fractures, allowing for adequate stability for the performance of simple activities of daily living and unloaded abduction. However, further research is necessary to investigate the biomechanical properties of PMMA as an orthopaedic restorative material and its potential for use in other clinical scenarios. With a better understanding of the mechanical characteristics and limitations of PMMA, it could be feasible to enhance this material for improved clinical outcomes in managing osteoporotic bone fractures.

## Data Availability

Data are contained within the article.
